# PBK/TOPK Expression Predicts Prognosis in Oral Cancer

**DOI:** 10.3390/ijms17071007

**Published:** 2016-06-24

**Authors:** Chin-Fang Chang, Sung-Lang Chen, Wen-Wei Sung, Ming-Ju Hsieh, Hui-Ting Hsu, Li-Hsin Chen, Mu-Kuan Chen, Jiunn-Liang Ko, Chih-Jung Chen, Ming-Chih Chou

**Affiliations:** 1 Institute of Medicine, Chung Shan Medical University, Taichung 402, Taiwan; benglung@hotmail.com (C.-F.C.); flutewayne@gmail.com (W.-W.S.); 128202@cch.org.tw (H.-T.H.); jlko@csmu.edu.tw (J.-L.K.); 2 Department of Otorhinolaryngology, Head and Neck Surgery, Jen-Ai Hospital, Taichung 400, Taiwan; 3 School of Medicine, Chung Shan Medical University, Taichung 402, Taiwan; cshy650@csh.org.tw (S.-L.C.); arsenelupinchen@yahoo.com.tw (L.-H.C.); 53780@cch.org.tw (M.-K.C.); 4 Department of Urology, Chung Shan Medical University Hospital, Taichung 402, Taiwan; 5 Department of Medical Education, Chung Shan Medical University Hospital, Taichung 402, Taiwan; 6 Department of Medical Technology, Jen-Teh Junior College of Medicine, Nursing and Management, Miaoli 356, Taiwan; 7 Cancer Research Center, Changhua Christian Hospital, Changhua 500, Taiwan; 170780@cch.org.tw; 8 School of Optometry, Chung Shan Medical University, Taichung 402, Taiwan; 9 Graduate Institute of Biomedical Sciences, China Medical University, Taichung 404, Taiwan; 10 Department of Surgical Pathology, Changhua Christian Hospital, Changhua 500, Taiwan; 11 Department of Otorhinolaryngology, Head and Neck Surgery, Changhua Christian Hospital, Changhua 500, Taiwan

**Keywords:** PBK (PDZ-binding kinase), TOPK (T-LAK cell-originated protein kinase), prognosis, head and neck cancer, metastasis, oral squamous cell carcinoma

## Abstract

Oral cancer is a common cancer with poor prognosis. We evaluated the expression of PBK/TOPK (PDZ-binding kinase/T-LAK cell-originated protein kinase) and its prognostic significance in oral cancer. PBK/TOPK expression was measured by immunohistochemical staining of samples from 287 patients with oral cancer. The association between PBK/TOPK expression and clinicopathological features was analyzed. The prognostic value of PBK/TOPK for overall survival was determined by Kaplan-Meier analysis and Cox proportional hazard models. A high PBK/TOPK expression level was correlated with long overall survival. The prognostic role of PBK/TOPK expression was significant in young patients (*p* < 0.05), patients with smoking habits (*p* < 0.05), and late stage disease (*p* < 0.05). Our results suggest that PBK/TOPK expression is enhanced in oral cancer. High PBK/TOPK expression, either alone or in subgroups according to clinicopathological features, may serve as a favorable prognostic marker for patients with oral cancer.

## 1. Introduction

Oral cancer is the major category of head and neck cancer and the sixth most common cancer worldwide. Globally, 400,000 new cases are reported each year, mostly from Asian countries [1]. In Taiwan, the mortality caused by head and neck cancer ranks fourth among males and sixth in the entire population [2].

Oral carcinogenesis is a multistep process that involves oncogenes and tumor suppressor genes via many factors, such as smoking, alcohol, pathogenic infections, and genetic factors [3,4]. Recent studies have also correlated the development of malignant tumors in the head and neck with a number of factors including inflammation, angiogenesis, and thrombosis [5]. Therefore, identification of specific biomarkers might provide useful information for clinical decision making and early prediction of prognosis in head and neck cancer especially the oral cancer.

PBK (PDZ-binding kinase), also known as T-LAK cell-originated protein kinase (TOPK), is a 322 amino-acid MAPKK-like serine/threonine kinase (mitogen-activated protein kinase kinase-like serine/threonine kinase) [6]. The PBK/TOPK protein is difficult to detect in normal tissues other than the germ cells of the testis and several fetal tissues [7]. Overexpression of PBK/TOPK was noted in activated T-LAK cells, where it interacted with hDlg (human homologue of the Drosophila Discs-large) through the C-terminal PDZ-binding motif of TOPK [8]. PBK/TOPK is highly expressed in various types of malignancies, such as malignant peripheral nerve sheath tumors, leukemia, breast cancer, and lymphoma [7,9,10,11]. PBK/TOPK over-expression contributes to tumor growth and proliferation in breast cancer, colorectal cancer, and Ewing sarcoma [6,7,10,12,13]. In lung cancer, patients with high expression of PBK/TOPK have poor clinical outcome [14]. In molecular studies, PBK/TOPK expression is correlated with cell apoptosis, inflammation, and mitotic regulation, and it may be regulated mainly via the c-Myc signaling pathway [15].

Accumulating evidence now indicates crucial roles for TOPK in cell-cycle regulation and tumorigenesis. However, the potential for an association between PBK/TOPK expression and oral squamous cell carcinoma has never been addressed. In this study, our aim was to evaluate the expression of PBK/TOPK and its clinical significance in oral squamous cell carcinoma.

## 2. Results

### 2.1. PBK/TOPK (PDZ-Binding Kinase/T-LAK Cell-Originated Protein Kinase) Is Detected in the Oral Cancer Specimens and Is Mainly Localized in the Cytoplasm

We verified the role of PBK/TOPK in the prognosis of oral cancer patients by recruiting 287 patients with primary tumors. The clinicopathological characteristics of the study subjects are listed in Table 1 and Table S1. The mean age was 55.92 ± 11.38 years (mean ± SD) with gender ratio of 0.21:1.00 (female:male). Though the habits of smoking, betel quid chewing, and alcohol consumption are considered as risk factors for oral cancer, in this study, the percentages of patients with these risk factors were relatively low (smoking, betel quid chewing, and alcohol consumption: 40.1%, 19.2%, and 39.4%, Table 1). In total, 114 patients had early stage tumors (stage I and II), and 173 patients had late stage tumors (stage III and IV).

**Figure 1:**
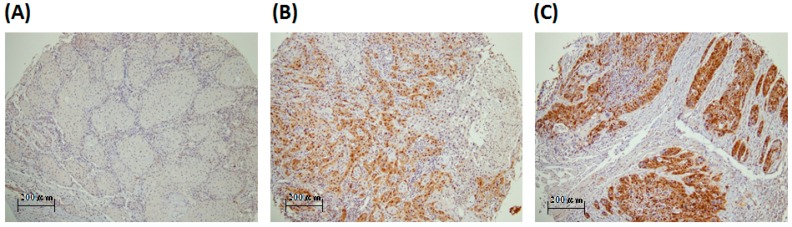
Representative immunostaining of PBK/TOPK in tissue arrays from patients with oral squamous cell carcinoma. PBK/TOPK expression scores were (**A**) 0 (stage 1); (**B**) 100 (stage 4); (**C**) >200 (stage 1), Scale bar = 100× (200 μm).

The PBK/TOPK (PDZ-binding kinase/T-LAK cell-originated protein kinase) expression was evaluated by IHC staining (Figure 1). PBK/TOPK expression level from peritumoral tissue and healthy candidates of representative whole section were also evaluated (Figure S1). The PBK/TOPK in oral squamous cell carcinoma was expressed in the nucleus or cytoplasm, appearing as brown or orange staining, and was difficult to detect in normal oral mucosa. The PBK/TOPK expression was scored separately by pathologists. All 287 specimens had both PBK/TOPK expression in the nucleus or cytoplasm. The mean PBK/TOPK expression score was 37.4 in the cytoplasm and 16.5 in the nucleus. We used cut-off points of 50 (upper quartile) for the cytoplasm and 10 (medium) for the nucleus in further analyses. Our analysis of revealed no significant correlation of PBK/TOPK expression with the clinicopathological variables of age, histological type, differentiation, lymph node metastasis, and TNM stage, whether in high or low cytoplasm PBK/TOPK-expressing tumors (Table 1).

### 2.2. The Prognostic Role of PBK/TOPK Expression in Oral Cancer Patients

**Figure 2:**
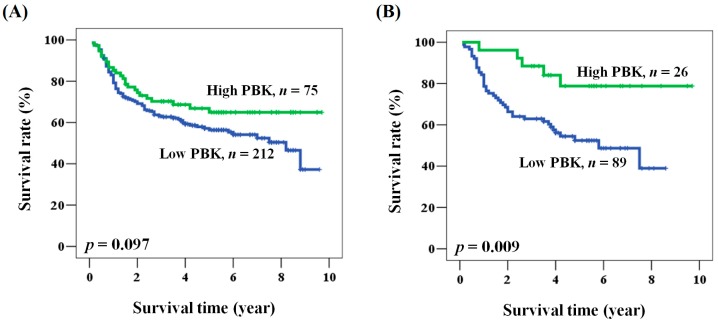
Kaplan-Meier analysis of PBK/TOPK expression with respect to the overall survival of (**A**) all patients and; (**B**) patients with smoking habits.

We sought further evidence for a prognostic role of PBK/TOPK expression in oral cancer patients. No data were missing among the 287 patients. The mean and median follow-up times after operation were 4.0 and 3.8 years (range from 0.1 to 9.7 years), respectively. The 5-year survival rate was 58.6%. During the survey, 168 (58.5%) patients died. Among the clinicopathological characteristics, advanced stage of the disease was significantly associated with poor clinical outcome (hazard ratio (HR) = 2.818, 95% CI = 1.841–4.313, *p* < 0.001 for univariate analysis; HR = 2.933, 95% CI = 1.900–4.529, *p* < 0.001 for multivariate analysis, Table 2 and Table 3). Patients with advanced stage cancer had shorter overall survival when compared with those with early stage disease (5-year survival: 47.0% vs. 75.7%, stage III + IV vs. I + II, *p* < 0.001). No significant association was noted between prognosis and clinicopathological characteristics other than stage (Table 2 and Table 3). A prognostic role for PBK/TOPK was suggested because overall survival was shorter for patients with low PBK/TOPK expression than for patients with high PBK/TOPK expression, although the difference was not statistically significant (Figure 2A). However, multivariate analysis showed that patients with low PBK/TOPK had a significantly poor prognosis (HR = 1.562, 95% CI = 1.002–2.435, *p* = 0.049 for multivariate analysis, Table 3).

### 2.3. Prognostic Role of PBK/TOPK Expression According to Clinicopathological Factors in Patients with Oral Cancer

We examined the prognostic role of PBK/TOPK in oral cancer patients by analyzing the clinical outcome according to clinicopathological factors. As shown in Table 4, multivariate analysis showed that PBK/TOPK expression was associated with overall survival rate (HR = 1.562, 95% CI = 1.002–2.435, *p* = 0.049). PBK/TOPK expression, when analyzed in the multivariate model, was significantly associated with the prognosis of patients of age <57, patients who smoked, and patients with moderately and poorly differentiated tumors, late pathological stage, or advanced *N* value. Kaplan-Meier analysis further supported the prognostic value of PBK/TOPK expression in oral cancer according to these characteristics (log rank *p* values: 0.014, 0.005, 0.012, 0.048, and 0.050 for patients of age <57, smoking, moderate and poor differentiation, late clinical stage, and advanced *N* value, respectively, Figure 2B). These results showed that the prognostic function of PBK/TOPK was significant with respect to specific clinicopathological characteristics.

## 3. Discussion

PBK/TOPK is a growth-factor-regulated kinase, which is constitutively high in tumor cells. Its expression is also induced in normal cells, but the ablation of PBK/TOPK does not affect normal cell cycle progression [7,8,9]. PBK/TOPK is phosphorylated and activated in a cell cycle-dependent manner while mitosis [8]. The site showing the most abundant expression is the placenta, whereas expression is absent or very low in normal adult brain tissue, a site where virtually no cell proliferation [8]. These results suggest that PBK/TOPK may have a role in the regulation of cell proliferation and cell cycle. Current studies implicate PBK/TOPK expression in tumor development, cancer growth, and apoptosis [6,9,10,14].

PBK/TOPK has been identified as prognostic markers for patients with leukemia, non-small cell lung cancer, colorectal cancer, and breast cancer [6,7,10,12,13,16]. High PBK/TOPK expression was associated with poor prognosis for cancer relapse, especially in lymph node-negative breast cancer patients [16]. Non-small cell lung cancer patients with high PBK/TOPK expression levels also had a poorer overall and recurrence survival when compared to patients with lower PBK/TOPK expression [14]. The present study is the first to report PBK/TOPK expression in oral cancer. Subjective bias was avoided by quantitative evaluation of PBK/TOPK expression using expression score measurements. We defined the expression score as the staining intensity of positive cells, calculated by a pathologist using a microscope; a higher score represented a higher level of PBK/TOPK expression. The present results confirmed that PBK/TOPK is over-expressed in oral cancer.

We also analyzed the correlation between PBK/TOPK expression and clinicopathological factors. The primary tumors of oral cancer showed no correlation between PBK/TOPK expression and the clinicopathological features of patient age, tumor histological type, differentiation, lymph node metastasis, and TNM stage. Univariate and multivariate analyses confirmed that the clinicopathological stage was significantly associated with poor prognosis and that PBK/TOPK expression was associated with good prognosis. These findings should be further confirmed with the mRNA expression of the specimens, which is our limitation. The expression of PBK/TOPK was correlated with young patient age (<57), smoking, moderate and poor differentiation, late pathological stage, and advanced N value. Investigation in other population would clarify the role of PBK/TOPK in the prognosis.

In conclusion, our study findings demonstrated that PBK/TOPK is specifically expressed in oral cancer and that increased expression is associated with good prognosis in some particular patients. The results support the suggestion that PBK/TOPK can serve as a valuable target for the development of tumor diagnosis and immunotherapy, although our findings need to be confirmed by further studies. Further molecular studies are also needed to provide a more in-depth picture regarding the function of PBK/TOPK in oral cancer.

## 4. Materials and Methods

### 4.1. Patients

The study was approved by the Institutional Review Board and the Ethics Committee of the Changhua Christian Hospital, Changhua, Taiwan (IRB No. 121008, 5 December 2012). Our study examined 287 samples of oral squamous cell carcinoma; these specimens were obtained from 114 patients with early stage (stage I and II) disease and 179 patients with late stage (stage III and IV) disease from the Department of Pathology of Chunghwa Christian Hospital. No patient was treated with chemotherapy or radiotherapy before surgical resection. The primary tumor patients were 237 males and 50 females, with a mean age of 55.92 years (range 31 to 90 years). These specimens were collected as tissue array as previous described [17,18]. For normal non-tumor oral mucosa, leukoplakia specimens from patients with leukoplakia found during routine oral cancer screen were used. The clinical parameters and overall survival data were collected at the time of database established from chart review and the Taiwan Cancer Registry, Department of Health, Executive Yuan, China. After that, the patients’ information was de-linked. Patient with never smoking, alcohol consumption, and betel quid chewing would be defined as negative in those habits. Clinicopathological features, including histological type, differentiation, lymph node metastasis, TNM stage, and tumor size, were assessed in this study. Histological diagnosis was confirmed by two pathologists. The median follow-up for overall survival was 3.95 years (range: 0.1 to 9.7 years).

### 4.2. Immunohistochemical Staining and Evaluation of PBK/TOPK Immunoreactivity

Immunohistochemical (IHC) staining was performed at the Department of Surgical Pathology, Changhua Christian Hospital, as previously described [16,17]. Anti-human PBK antibody (GAP1 antibody, A-6, 1:50 dilution; sc-271110, Santa Cruz Biotechnology, Santa Cruz, CA, USA) was used, as described in previous studies [19,20]. The sections were placed on coated slides, deparaffinized with xylene, and rehydrated through serial dilutions of alcohol, followed by washings with phosphate buffered saline (PBS) (pH = 7.2). Endogenous peroxidase activity was blocked with 3% H_2_O_2_. Antigen retrieval was performed by boiling in citrate buffer (10 mM) for 20 min. The sections were incubated with an anti-human PBK antibody for 20 min at room temperature and then thoroughly washed. The immunoreaction was visualized using a polymer-based MACH4 DAB Detection Kit (Biocare Medical, Concord, CA, USA) according to the manufacturer’s instructions to obtain optimal immunoreactivity and the fewest background artifacts. The slides were incubated with a horseradish peroxidase (HRP)/Fab polymer conjugate for another 30 min. The sites of peroxidase activity were visualized using 3,3′-diamino-benzidine tetrahydrochloride (Biocare Medical, CA, USA) as the substrate for 5 min and counterstained with hematoxylin (Biocare Medical, CA, USA). PBS was used instead of primary antibodies as a negative control. Immunoreactivity scores were analyzed by three pathologists, using scores defined as previously described [18,21]. The pathologists were blinded to the prognostic data of this study. A final agreement was obtained for each score by viewing through a multiheaded microscope (Olympus BX51 10 headed microscopes, Olympus, Tokyo, Japan). Briefly, immunoreactivity scores were defined as the cell staining intensity (0 = nil; 1 = weak; 2 = moderate; and 3 = strong) multiplied by the percentage of stained cells (0%–100%), leading to scores from 0 to 300. The expression score was calculated as [percentage of stained cells × staining intensity (0–3)].

### 4.3. Statistical Analysis

The χ^2^ test was applied for continuous or discrete data analysis. The associations between PBK/TOPK expression and patient survival were estimated using univariate analysis and the Kaplan-Meier method and assessed using the log-rank test. Potential confounders, including age, gender, and stage, were adjusted by Cox regression models of multivariate analysis, with the PBK/TOPK expression fitted as indicator variables. Overall survival time was defined as the interval between the date of surgery and the date of the last follow-up or death. All statistical analyses were conducted using SPSS statistical software (version 15.0) (SPSS, Inc., Chicago, IL, USA). All statistical tests were 2-sided, and the values of *p* < 0.05 were considered statistically significant.

## Figures and Tables

**Table 1:** Relationships between PBK/TOPK (PDZ-binding kinase/T-LAK cell-originated protein kinase) expression and clinical parameters in 287 oral cancer patients.

Parameters	Case Number	PBK/TOPK Expression		*p* Value
		Low	High	
Age (year)		55.5 ± 11.2	57.1 ± 11.9	0.292
Gender				
Female	50	36 (72.0)	14 (28.0)	0.741
Male	237	176 (74.3)	61 (25.7)	
Smoking				
No	172	123 (71.5)	49 (28.5)	0.267
Yes	115	89 (77.4)	26 (22.6)	
Betel quid chewing				
No	232	169 (72.8)	63 (27.2)	0.418
Yes	55	43 (78.2)	12 (21.8)	
Alcohol consumption				
No	174	130 (74.7)	44 (25.3)	0.686
Yes	113	82 (72.6)	31 (27.4)	
Differentiation				
Well	43	37 (86.0)	6 (14.0)	0.096
Moderate	237	171 (72.2)	66 (27.8)	
Poor	7	4 (57.1)	3 (42.9)	
Stage				
I + II	114	87 (76.3)	27 (23.7)	0.443
III + IV	173	125 (73.0)	48 (27.7)	
*T* value				
1 + 2 + 3	189	138 (73.0)	51 (27.0)	0.648
4	98	74 (75.5)	24 (24.5)	
*N* value				
0	174	134 (77.0)	40 (23.0)	0.133
1 + 2 + 3	113	78 (69.0)	35 (31.0)	

**Table 2:** Univariate analysis of the influence of clinicopathological parameters on the overall survival of oral cancer patients.

Parameter	Category	Overall Survival			
		5-Year Survival (%)	HR	95% CI	*p* Value
Age, year	≥57/<57	60.4/59.2	1.043	0.726–1.499	0.820
Gender	Male/Female	57.7/69.9	1.496	0.870–2.572	0.145
Smoking	Yes/No	58.4/58.7	0.951	0.657–1.375	0.789
Betel quid chewing	Yes/No	59.4/58.1	0.846	0.523–1.370	0.497
Alcohol consumption	Yes/No	56.0/60.5	1.119	0.775–1.614	0.548
Stage	III + IV/I + II	47.0/75.7	2.818	1.841–4.313	<0.001
PBK/TOPK	Low/High	56.3/64.9	1.447	0.930–2.250	0.101

**Table 3:** Multivariate analysis of the influence of clinicopathological parameters on the overall survival of oral cancer patients.

Parameter	Category	Overall Survival *			
		5-Year Survival (%)	HR	95% CI	*p* Value
Age, year	≥57/<57	60.4/59.2	0.942	0.651–1.364	0.753
Gender	Male/Female	57.7/69.9	1.412	0.797–2.503	0.237
Smoking	Yes/No	58.4/58.7	0.957	0.613–1.496	0.849
Betel quid chewing	Yes/No	59.4/58.1	0.763	0.431–1.350	0.352
Alcohol consumption	Yes/No	56.0/60.5	0.907	0.618–1.330	0.616
Stage	III + IV/I + II	47.0/75.7	2.933	1.900–4.529	<0.001
PBK/TOPK	Low/High	56.3/64.9	1.562	1.002–2.435	0.049

* Adjusted for age, gender, smoking, betel quid, alcohol consumption, and stage.

**Table 4:** Multivariate analysis of the PBK/TOPK expression according to clinical parameters in overall survival of patients with oral cancer.

Parameter	Overall Survival ^1^			
	5-Year Survival (%) ^2^	HR	95% CI	*p* Value
All cases	56.3/64.9	1.562	1.002–2.435	0.049
Age (year)				
<57	54.9/71.9	2.276	1.182–4.380	0.014
≥57	58.7/56.1	0.950	0.504–1.791	0.874
Gender				
Female	64.3/84.4	3.037	0.632–14.595	0.166
Male	54.9/61.1	1.442	0.904–2.298	0.124
Smoking				
No	59.2/57.6	0.976	0.579–1.646	0.929
Yes	52.4/78.8	3.896	1.515-10.017	0.005
Betel quid chewing				
No	56.1/63.8	1.428	0.888–2.297	0.142
Yes	56.5/71.3	2.821	0.795–10.006	0.108
Alcohol consumption				
No	57.1/71.0	2.029	1.083–3.804	0.027
Yes	55.7/56.4	1.109	0.575–2.139	0.757
Differentiation				
Well	78.2/55.6	0.980	0.153–6.272	0.983
Moderate + Poor	51.7/65.5	1.813	1.141–2.880	0.012
Stage				
I + II	75.7/76.7	1.213	0.480–3.063	0.683
III + IV	42.8/58.0	1.680	1.005–2.810	0.048
*T* value				
1 + 2 + 3	62.1/71.3	1.808	0.991–3.298	0.054
4	45.8/50.7	1.297	0.649–2.591	0.462
*N* value				
0	69.1/76.4	1.644	0.797–3.394	0.179
1 + 2 + 3	34.0/51.4	1.762	1.000–3.104	0.050

^1^ Adjusted for age, gender, smoking, betel quid chewing, alcohol consumption, and stage; ^2^ PBK/TOPK expression: Low/High.

## Supplementary Materials

Supplementary materials can be found at http://www.mdpi.com/1422-0067/17/7/1007/s1.
