# Supplementary material for: PBK/TOPK Expression Predicts Prognosis in Oral Cancer
Source: Int J Mol Sci. 2016 Jun 24;17(7):1007. doi: 10.3390/ijms17071007 (PMC4964383; doi:10.3390/ijms17071007)
Supplement: Supplementary file 1 [file ijms-17-01007-s001.pdf]

Supplementary Materials: PBK/TOPK Expression Predicts Prognosis in Oral Cancer

Chin-Fang Chang, Sung-Lang Chen, Wen-Wei Sung, Ming-Ju Hsieh, Hui-Ting Hsu, Li-Hsin Chen, Mu-Kuan Chen, Jiunn-Liang Ko, Chih-Jung Chen and Ming-Chih Chou

Table S1. Detail information of stage, *T* value, and *N* value according to PBK/TOPK expression in 287 oral cancer patients.

Parameters	Case Number	PBK/TOPK Expression		*p* Value
		Low	High	
Stage				
I	61	47 (77.0)	14 (23.0)	0.823
II	53	40 (75.5)	13 (24.5)	
III	37	28 (75.7)	9 (24.3)	
IV	136	97 (71.3)	39 (28.7)	
T value				
1	80	60 (75.0)	20 (25.0)	0.846
2	88	64 (72.7)	24 (27.3)	
3	21	14 (66.7)	7 (33.3)	
4	98	74 (75.5)	24 (24.5)	
N value				
0	174	134 (77.0)	40 (23.0)	0.072
1	41	33 (80.5)	8 (19.5)	
2	68	42 (61.8)	26 (38.2)	
3	4	3 (75.0)	1 (25.0)	

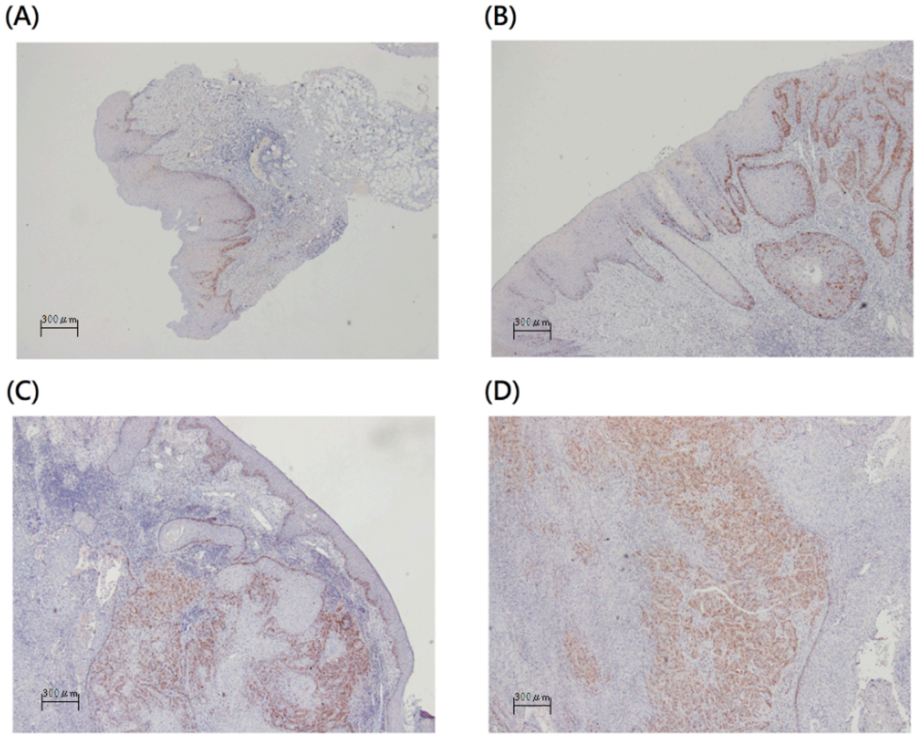

Figure S1. The representing IHC staining of PBK/TOPK in a healthy candidate (oral leukoplakia) (**A**), and three tumor whole sections (**B**–**D**). Clinical information: (**B**) T3N0M0, follow up duration: 7.0 years, alive, score: 20; (**C**) T3N2M0, follow up duration: 4.0 years, dead, score: 60; (**D**) T3N0M0, follow up duration: 8.4 years, alive, score: 75. Scale bar = 40× (300 μm)
